# Literary Notes

**Published:** 1907-01

**Authors:** 


### LITERARY NOTES.

THE *Long Island Medical Journal* is the title of a new local publication, edited by Dr. Paul M. Pilcher, and published under the auspices of the Associated Physicians of Long Island. This journal, says the Brooklyn Medical Journal, will in a great measure take the place of the Brooklyn Medical Journal, which ceases publication with the December issue. It will cover all the Island news, reporting transactions of all the county societies, and in addition give full accounts of the meetings of the different sections of the Kings County Medical Society. It will be almost distinctively a local journal, and as such should receive generous support. The first number will be published in January, 1907, and will appear regularly the fifteenth of each succeeding month.

---

The *Brooklyn Medical Journal* announces in its issue for December, 1906, that its publication ceases with that number. It has been published for twenty years and is the kind of journal from which we regret to part. As the official organ of the Medical Society of the County of Kings it has proved itself not only loyal to the interests of the society, but has taken high place in the journalistic literature of the country.

---

The *Oklahama Medical News Journal,* beginning with January, 1907, will be edited by Dr. Y. E. Colville of Chattanooga, Tenn. Dr. Colville has purchased a half interest in the magazine and will devote his entire time to the editorial department, while Dr. Phelan will continue as business manager. It is announced that the journal will be considerably enlarged as well as otherwise improved.

---

The *Gazette Médicale de Paris,* the oldest medical journal in France, will henceforth be published under the editorship and management of Dr. Lucien-Graux, who is also editor in chief of the *Gazette des Eaux.*

